# The Development of a Smart Health Awareness Message Framework Based on the Use of Social Media: Quantitative Study

**DOI:** 10.2196/16212

**Published:** 2020-07-23

**Authors:** Elaf Ali Alsisi, Ahmed Al-Ashaab, Wadhah Ahmed Abualfaraa

**Affiliations:** 1 Cranfield University Cranfield United Kingdom

**Keywords:** social media, health promotion and social media, health awareness, health promotion, eHealth, technology acceptance theory

## Abstract

**Background:**

Social media has recently provided a remarkable means of delivering health information broadly and in a cost-effective way. Despite its benefits, some difficulties are encountered in attempting to influence the public to change their behavior in response to social media health messages.

**Objective:**

This study aimed to explore the factors that affect individuals’ acceptance of using social media as a tool for receiving health awareness messages and adapting such content accordingly by developing a smart health awareness message framework.

**Methods:**

A quantitative method was adapted to validate the hypotheses and proposed framework through the development of a survey based on the technology acceptance model with the extension of other constructs. The survey was distributed on the web to 701 participants from different countries via Qualtrics software; it generated 391 completed questionnaires, and the response rate was 55.8% (391/701).

**Results:**

Of the 391 respondents, 121 (30.9%) used social media platforms often during the week, and 27 participants (6.9%) did not use social media. In addition, 24.0% (94/391) of the respondents used these platforms to seek health information. On the basis of the results, perceived usefulness (β=.37; *P*<.001), gain-framed message (β=.04; *P*<.001), and loss-framed message (β=.08; *P*<.001) were seen to positively and significantly influence people’s intention to use social media as a means to spread information about health promotion. The proposed smart health awareness message framework identifies 64.2% of the variance in intention to use, 55.4% of the variance of perceived usefulness, and 26.2% of the variance of perceived ease of use.

**Conclusions:**

This study sheds light on the factors that are associated with people’s intention to use and adopt social media in the health promotion domain. The findings reveal that the intention of using social media for health awareness purposes is positively impacted by the perception of usefulness of social media and the design of health messages. Future research might seek to explore other factors that relate to people’s behavior. This point of view will assist health organizations in developing their health messages more effectively and to be patient friendly.

## Introduction

### Background

The advent of the internet has become a fundamental avenue for gaining health information [1] and for the provision of interventions to enhance healthy behaviors [2]. Rapid and easy access to the internet has driven individuals to go on the web to seek health information [3]. The internet has contributed to the evolution of a new era of communication, known as social media. The phenomenon of social media is defined as a collection of web-based social networking apps that enable individuals or groups of people to communicate and interact with each other, share information, collaborate, and exchange content [4,5]. The advancement of the internet, web-based health information, and social media has driven the emergence of electronic health (eHealth). According to the World Health Organization [6], eHealth can be defined as “the use of information and communication technologies (ICT),” which involves the development of spreading health information through digital methods [7,8].

One aspect of public health communication, which has received increasing attention, is the media channels through which health messages could be successfully conveyed to a wide range of relevant audiences. Several studies have found that mass media (eg, television, radio, newspaper, leaflets, and posters) have a positive impact on health promotion [9-12]. However, others have marked the ineffectiveness of this impact [13], unlike social media, which has greater potential in health promotion for individuals and health care professionals because of its ability to deliver meaningful health content [14] in different formats such as text, images, and videos [15]. It can be argued, therefore, that social media has largely proved to be an effective and influential instrument in spreading health awareness messages [16] because of its easy access by all socioeconomic strata and its cost effectiveness [3]. Due to the evolution of eHealth and social media tools, health organizations reinforce practitioners in providing health-related information to increase health awareness and obtain better health outcomes [17,18].

Despite the increasing utilization of social media by health organizations in disseminating health awareness, the actual impact of social media interventions demands further research to explore the factors that may affect users’ acceptance of this technology and adoption of the content [15,19]. These factors include the frame of the message, trust of the content, and the degree of technology acceptance [20-22]. This study is motivated by the need to take into consideration such key factors that lead to effective acceptance of social media as a means to receive, read, and apply health awareness messages.

The paper provides the results of the smart health awareness message framework development and, in turn, ensures spreading health awareness messages effectively on a faster and wider scale through social media. The focus of this paper presents the identification of the factors influencing an individual’s intention to use social media as a means to receiving health awareness messages and following its instructions for the well-being of the individual by using the technology acceptance model (TAM) [23], task technology fit (TTF) [24], and prospect theory [25].

The research approach starts with a review of related literature concerning health awareness messages and the use of social media in spreading such messages to a wider community. The second stage involved developing a conceptual framework of the factors influencing an individual’s intention to use social media for health promotion. The effectiveness of the proposed framework was evaluated based on the hypotheses developed in this study. To validate these hypotheses, public opinion was analyzed based on a web-based survey using the Qualtrics software with 391 participants.

Such a random sample size would be a good representative because it reflects the characteristics of the population from which it has been drawn (ie, from a wide range of countries) and different opinions that were relatively close to each other.

The remainder of this paper is structured as follows. The first section includes an introduction that presents the research motivation, research approach, and the aim of this paper. The second section presents a review of the related literature. The third section presents the conceptual framework along with the proposed hypotheses. In the fourth section, methods of data collection and measurement development are presented. The section following the fourth section presents some public perspectives of the smart health awareness message framework through data analysis, including testing hypotheses. Finally, the authors conclude with a discussion of the research limitations and future work.

### Literature Review

Public health communication has emerged as a modern strategy to change public behavior by raising awareness of risk diseases. Public health communication refers to “the scientific development, strategic dissemination, and critical evaluation of relevant, accurate, accessible, and understandable health information communicated to and from intended audiences to advance the health of the public” [26]. Therefore, health promotion encompasses the development of approaches that supply health knowledge to individuals, motivating them to adopt the healthy behaviors and change their current ones [27]. Traditionally, mass media has been used as a tool for public health promotion, which has involved a variety of forms including television, newspapers, radio, booklets, billboards, leaflets, and posters [9,12,28]. Each format varies according to the level of effectiveness and drawbacks. For example, numerous studies have explored the efficacy of using television campaigns to promote smoking cessation [29,30]. However, exposure to such campaigns has been found to be expensive in comparison with radio broadcasts [12,30].

Although several studies have highlighted the effectiveness of promoting health awareness via leaflets and posters [31-33], the reality is that they are still an expensive media to be published. This is due to the long process and expense of publishing paper-based media and also the factors such as time and labor consumption, limited information being given to the audience [34], poor health content [35], and overlapping information [36]. These reservations also include editing the health content, graphical design, printing, and distributing. Updating any of these printed media requires a further long loop of modification.

Social media has a great potential in public health communication, as it provides patients and the public with the best opportunity by delivering meaningful health content. Ba and Wang [14] found that online social groups have an essential role to play in an individual’s routine in terms of encouraging them to adopt a healthy lifestyle through observing their daily diet. Previous research has focused on customized digital health interventions that help individuals to control chronic disease and make proper decisions accordingly [37]. Roland et al [38] developed an online community represented by #FOAMed on Twitter for the purpose of sharing medical knowledge. Similarly, Diddi and Lundy [39] indicated the usage of Twitter to spread breast cancer awareness by 4 different health organizations, presenting different factors of the health belief model in the content of the message. A previous study has supported diabetic people by offering a forum for sharing personal experience and providing feedback on performance by physicians [40].

So far, few research studies have examined the influential factors that affect people’s intention to use social media in the health promotion context [15,20]. However, understanding these factors is important for designing health promotion messages that incorporate content strategy and simplicity [41]. To fill this research gap, a conceptual smart health awareness message framework was developed based on the TAM, TTF, and prospect theory, as presented in the following section.

The TAM assumes that the extent to which the technology is accepted and used by an individual is predicted by 2 main constructs (factors): perceived usefulness and perceived ease of use [23]. TTF focuses mainly on the features that the technology offers, and thus, it believes that technology must match the task it supports to have performance impact [24]. TTF has 4 key constructs (elements), one of which is technology characteristics. Prospect theory postulates that health communication messages can be designed to shed light on the benefits (gain) or the consequences (loss) of performing a specific behavior [25].

### Smart Health Awareness Message Framework and Hypothesis Development

The smart health awareness message framework includes different elements, which are called constructs, and each construct represents the key factor of a different adapted theory. Thus, this study investigates the impact of such constructs that influence an individual’s acceptance of using social media as a tool for receiving health awareness messages and consequently following its instructions for the individual’s well-being. The authors adapted the key constructs of 3 theoretical foundations: (1) TAM, (2) TTF, and (3) prospect theory. The TAM serves as a concrete base to develop the conceptual framework. The TTF offers a key element of social media characteristics, whereas the prospect theory provides a theoretical framework for designing such messages. The proposed framework, therefore, will help in designing health messages that will be spread via social media apps.

**Figure 1:**
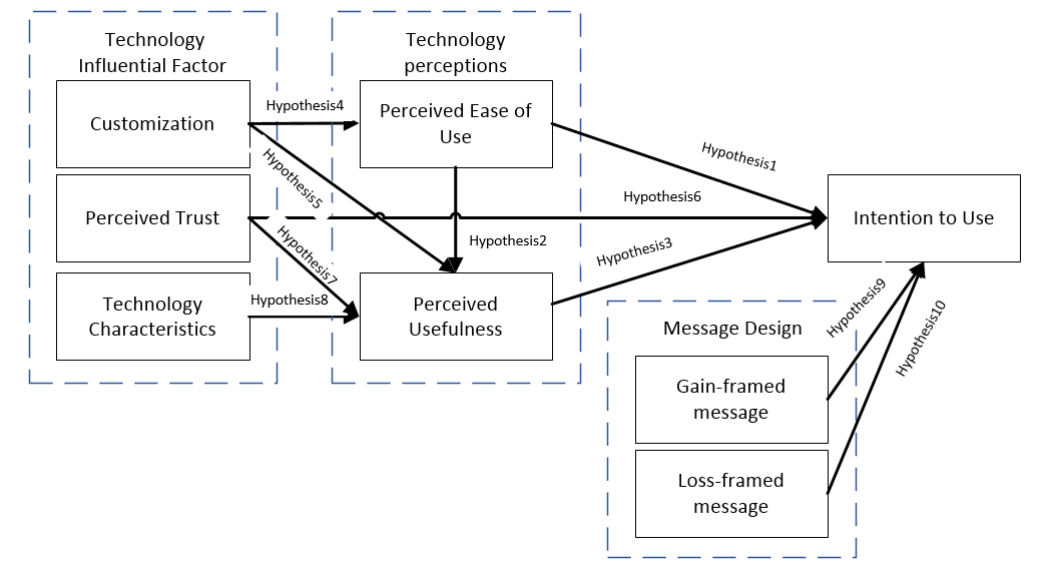
Smart Health Awareness Message Framework.

Figure 1 illustrates smart health awareness message framework, where the authors proposed different hypotheses that provide a statement based on the feature extracted from the intended theory to represent a specific state of an individual’s beliefs. This is to be used in a survey to obtain public perspectives on the use of social media technology in receiving health messages. First, they hypothesized that intention to use is influenced by perceived ease of use, perceived usefulness, perceived trust, gain-framed message, and loss-framed message (hypotheses H1, H3, H6, H9, and H10). Second, the authors hypothesized that perceived usefulness is impacted by perceived ease of use, customization, perceived trust, and technology characteristics (H2, H5, H7, and H8). Finally, it was hypothesized that perceived ease of use is influenced by customization (H4). Each defined hypothesis supports the relationships among the constructs of the framework. The following subsections present in detail the constructs of smart health awareness message framework.

#### Technology Perceptions

Technology perceptions include the key elements of the TAM, namely, perceived ease of use and perceived usefulness.

Perceived ease of use, as proposed by Davis [23], alludes to the extent to which the user of the technology will think that the use of a certain tool will be easy or free of effort. As social media supports easy access, easy engagement with its interactions (ie, reply, like, retweet, and repost) [42,43], and easy navigation, people’s intention will be enhanced to receive and apply health messages accordingly [44]. Exposure to such interactions is generally dynamic, resulting in encouraging health care professionals to create online communities where medical knowledge can be easily shared and freely accessed [38].

A positive association was supported between perceived ease of use and usefulness of technology usage that involved different contexts [45-47]. Therefore, H1 states that perceived ease of use of social media positively influences people’s intention to receive and follow health awareness messages. H2 states that perceived ease of use positively influences perceived usefulness of social media to receive and follow health awareness messages.

Perceived usefulness is widely defined as ‘‘the degree to which an individual believes that using a particular system would enhance his/her job performance’’ [23]. In this study, perceived usefulness refers to the degree to which an individual considers that the benefits of social media will enhance his or her intention to receive and then follow health awareness messages. Some individuals may perceive social media as a personal digital assistant with the purpose of improving medical usage [48].

The link between the usefulness of social media and the intention to adopt such technology as a means to acquire and share health information has been explored by a number of studies [47,49]. Moreover, Deng et al [50] investigated the association between perceived usefulness and the individual’s intention to adapt to mobile health. Therefore, H3 states that perceived usefulness of using social media will positively influence people’s intention to receive and follow health awareness messages.

#### Technology-Influencing Factors

This section includes 3 technology factors, customization, perceived trust, and technology characteristics, which influence the overall perceptions of social media to receive and follow health awareness messages.

Message customization means reaching target people with individualized health messages that work well to engage with the messages effectively [51]. Patrick et al [52] found that people who were exposed to customized text messages with the purpose of promoting dietary behaviors were more likely to achieve weight loss compared with those related to printed materials. Customization in the conceptual framework refers to the empowerment that enables intended systems to understand its users’ demographics and interest topics and then tailor their health messages accordingly, for example, the preferable time, the frequency of the messages, the type of disease, and the type of social media platforms. Social media customization provides a number of features that encourage users to prioritize particular accounts to view and act accordingly. On Twitter, Instagram, Facebook, and WhatsApp, by selecting get notifications, a user will automatically be notified every time these accounts post. Thus, such features allow users to easily access and efficiently follow social media posts. By leveraging social media customization efficiently, such technology can be harnessed to be most instrumental and useful in practice.

Customization correlates to perceived usefulness, as evidenced by Ho [53], who found that customized information technology services offer considerable benefits to customers that involve producing right content and format at the proper time for usage motivation purposes. In addition, customization in web-based interfaces has optimized web-based shopping due to its ease of use [54]. Therefore, H4 states that customization will have a positive impact on perceived ease of use of social media platforms to receive and follow health awareness messages. H5 states that customization will have a positive impact on perceived usefulness of social media platforms to receive and follow health awareness messages.

McAllister [55] defined trust as “the extent to which a person is confident in, and willing to act on the basis of, the words, actions, and decisions of another.” He studied the trust between patients and the source of health-related information. Undoubtedly, the trust that individuals place in web-based health knowledge varies with the source of such knowledge. Thus, trust is a significant factor that affects people’s adoption of health awareness messages [56,57]. Perceived trust in the framework will evaluate the contextual part of health-related information, and perceived usefulness will evaluate the practical part of social media use for passing health messages.

The association between trust and perceived usefulness has been discussed in several studies, confirming that the more the user perceives the technology to be useful, the greater the likelihood of trusting the content of such technology and therefore their intention to use it [58-60]. Thus, H6 states that perceived trust will positively influence people’s intention to use social media to receive and follow health awareness messages. H7 states that perceived trust will positively impact perceived usefulness of social media to receive and follow health awareness messages.

Technology characteristics constitute a key element of the TTF model identified by Goodhe and Thompson [24], which refers to the extent to which a technology fits when the required tasks are met. The more individuals perceive that the technology suitably fits the intended tasks, the greater the likelihood that they will use that technology [61]. TTF has been used to measure social media appropriateness in many topics, such as sharing information about flood anticipation [62]. A previous study proposed that sharing is a fundamental characteristic that social media provides [63], which refers to the extent to which content can be exchanged among users [64]. For Facebook, Twitter, and Instagram, qualities that correspond to sharing are the message itself and media representation, which involves photographs and videos in terms of photograph quantity and video length [22].

The suitability of TTF depends on the user selection of the technology, which is based on technology characteristics that perfectly correspond to the task’s attributes. Hence, this research demonstrates that social media features are capable of boosting the adaptation of this technology in viewing health awareness messages. Earlier studies have investigated the relationship between TTF constructs and perceived usefulness of using SMS for health awareness purposes [65]. Therefore, H8 states that technology characteristics will positively impact perceived usefulness of social media to view health awareness messages.

#### Message Design

This section presents a technique that aids in designing health awareness messages through prospect theory.

In loss- and gain-framed message design, health messages that aim at a particular behavior in terms of its benefits (gains) or costs (losses) play a significant role in health communication [57,66]. Health messages, therefore, might be designed either to emphasize the benefits of complying with the message content or the consequences of failure to comply with it [67,68]. For instance, a gain-framed message targeting increasing water intake could be *drinking a lot of water daily can help you lose weight*. In contrast, a loss-framed message could be *not drinking enough water causes you to gain weight* [69]. According to the prospect theory, the associated persuasion of gain- and loss-framed appeals is linked to the level of risk involved in the relevant actions [25]. The more individuals believe that they are at risk, the more motivated they are to the loss-framed message [57]. Therefore, the effectiveness of gain- and loss-framed messages varies depending on the goal of the message, either preventing or detecting health problems [66,70]. Therefore, positive messages are manifested to be more powerful in disease prevention messages [68], such as skin cancer prevention [66], whereas loss-framed messages are more likely to be useful in disease detection [71]. A number of studies have examined the effectiveness of negative messages in designing persuasive health messages. Meyerowitz and Chaiken [71] investigated the issue of women’s breast self-assessment, indicating that female students were more encouraged to perform the assessment through passive messages rather than positive ones. Levin et al [72] developed a framing effect-based typology to check the influence of negative- and positive-framed information on decision makers. They concluded that passive goal framing was more convincing and influential than positive ones.

Message frame is believed to have a significant relationship with an individual’s intention to adapt to technological invention [73]. Hence, H9 states that the positive effect of gain-framed messages on consumers ‘intention to use social media for health awareness purposes would be stronger. On the other hand, hypothesis 10 states that the positive effect of loss-framed messages would be stronger.

## Methods

### Data Collection

The authors developed the questionnaire items based on an understanding of the literature, as presented in Multimedia Appendix 1. Before conducting the survey, it was validated by 5 experts in different industrial and research fields, including community medicine consultants, family medicine consultants, oncologists, and public health specialists based in Saudi Arabia, the United Kingdom, and the United Arab Emirates. Including qualified experts’ opinions will assure that items are clarified, accurate, and free of confusion. The survey was distributed on the web through Qualtrics over a 2-month period in 2019, and it produced 701 responses from different countries, with 391 completed surveys.

### Development of Questionnaire Items

The questionnaire included 3 parts: the first part presented the survey’s introduction and consent form, the second part focused on the participant’s demographics, as shown in Table 1, and the third part included 27 items. Each item is a statement that has been adapted from the literature to measure the opinion of the end user regarding the 7 identified constructs of the conceptual framework. List of items are presented in Multimedia Appendix 1; both items and scales were adapted from previous studies with some modifications to fit the research context. Perceived ease of use and intention to use social media items were adapted from Hong et al [74]. The items of perceived usefulness on the intention to use social media were borrowed from El-Wajeeh et al [75]. Items on customization were adapted from Bandyopadhyay et al [65]. The items on the perceived trust construct were adapted from El-Wajeeh et al [75], and items on technology characteristics were derived from Bandyopadhyay et al [65] and Zaini et al [76]. All questionnaire items were rated using a 5-point Likert scale, ranging from *strongly disagree* (1) to *strongly agree* (5), in which participants were required to choose the most suitable answer.

**Table 1:** Respondent demographics (N=391).

Measure		Values, n (%)	
**Gender**			
	Male		154 (39.4)
	Female		237 (60.6^a^)
**Age (years)**			
	20-29		91 (23.3)
	30-39		142 (36.3^a^)
	40-49		64 (16.4)
	50-59		52 (13.3)
	≥60		42 (10.7)
**Level of education**			
	Secondary school		9 (2.3)
	Bachelor’s degree		125 (32.0)
	Master’s degree or above		227 (58.1^a^)
	Others		30 (7.7)
**Job**			
	Governmental employee		184 (47.1^a^)
	Private employee		85 (21.7)
	Self-employed		21 (5.4)
	I do not work		101 (25.8)
**Frequency of using social media during the week**			
	Always		74 (18.9)
	Very often		75 (19.2)
	Often		121 (31.0^a^)
	Hardly often		94 (24.0)
	Never		27 (6.9)
**Frequency of using social media for seeking health information (years)**			
	<2		121 (31.0^a^)
	2 to <4		84 (21.5)
	4-6		94 (24.0)
	>6		92 (23.5)

^a^Indicates the highest percentage.

### Respondent Profile and Descriptive Statistics

The respondents’ demographics are illustrated in Table 1. Of the 391 participants, 121 used social media platforms often during the week, with a percentage of 30.9% (Table 1). In total, 18.9% (74/391) and 19.2% (75/391) of the participants used them always and very often during the week, respectively (Table 1). Conversely, 6.9% (27/391) of the participants never used social media (Table 1). In addition, 24.0% (94/391) of the respondents utilized these platforms to seek health information (Table 1). This is due to several reasons, including easy and free access to social media, with no physical existence requirement as with health care centers, and no storage capacity is needed as with printed media.

## Results

### Data Analysis

Smart health awareness message framework has been proposed to elicit the opinion of the end user about different constructs, and the results of the survey require a range of statistical methods. First, SPSS (version 25; IBM Corp) was used to acquire respondents’ descriptive statistics. Then, data were analyzed using the IBM SPSS Analysis of a Moment Structures (AMOS) version 25, which requires 2 stages of assessment: measurement model assessment and structural equation modeling (SEM) assessment. The measurement model was assessed to confirm that the survey items reflected the corresponding constructs of the conceptual framework [77]. SEM was used to test hypothesized relationships among the constructs after conducting confirmatory factor analysis (CFA). The following subsections present the results of exploratory factor analysis (EFA), which includes a measurement model followed by SEM.

### Measurement Model

In the first stage, an EFA was conducted to determine the correlation among observed variables or items being tested. A correlation matrix presented in Multimedia Appendix 2 shows the internal correlations between variables, which are higher than ±0.3, and not exceeding the cut-off threshold, ±0.8, refers to the absence of multicollinearity [77]. Then, EFA was conducted and provided a factor structure of 27 items (Multimedia Appendix 1). These variables are grouped into 7 factors: perceived ease of use, perceived usefulness, customization, perceived trust, technology characteristics, gain- and loss-framed message, and intention to use. The factor analysis results are illustrated in Table 2 using maximum likelihood with a promax rotation of data. This analysis shows a clean factor loading pattern, no major cross loading, where values ranged between 0.3 and 0.8, cut-off criteria [78].

Another issue to be considered in EFA is the appropriateness of the data set that has been verified using the Kaiser-Meyer-Olkin (KMO) statistics and Bartlett test of sphericity. According to Kaiser [79], the KMO value is recommended to be greater than 0.7 to obtain meaningful and good EFA. To assure the factorability of the correlation matrix among variables, the Bartlett test value should be significant (*P*<.01) [80]. The KMO yielded data adequacy with a value of 0.80, and the sphericity test showed a statistically significant χ^2^_190_=2467.0 (*P*<.01; Multimedia Appendix 3). Thus, it is evident that the factorability of the correlation matrix is adequate. Then, construct reliability was measured by Cronbach alpha (CA), composite reliability (CR), and average variance extracted (AVE). CA was .893 for the total items; thus, the value was greater than the recommended .7 [78]. Table 3 presents CA for each construct, ranging between .733 and .826, leading to fit reliabilities of the data. Convergent validity can be assessed by calculating the average variance extracted and CR where the values should be greater than 0.5 and 0.7, respectively [81]. The results in Table 3 reveal that the AVE and CR applied such criteria. Although the AVE of technology characteristics is below the recommended value, Fornell and Larcker [82] confirmed that a researcher may conclude that the convergent validity of the construct is adequate, as CR is higher than the acceptable range.

Discriminant validity refers to the extent to which the constructs are varied from each other, which can be assessed using the Fornell-Larcker criterion [82]. In this method, the square root of AVE is compared with the correlation of constructs or variables. The variance between constructs and their items should exceed the variance explained with other constructs [82]. Table 4 illustrates that all diagonal square roots of the AVEs were higher than the off-diagonal values, which present constructs’ correlations. Given the adequate reliability and acceptable convergent and discriminant validities, it is concluded that the measurement model is satisfactory.

**Table 2:** Promax matrix showing factor analysis results.

Factor^a,b^	1	2	3	4	5	6	7
	PU ^c^	PEU^d^	PT^e^	TECH^f^	CUST^g^	INT^h^	Message^i^
PU1	0.406	N/A^j^	N/A	N/A	N/A	N/A	N/A
PU2	0.512	N/A	N/A	N/A	N/A	N/A	N/A
PEU1	N/A	0.789	N/A	N/A	N/A	N/A	N/A
PEU2	N/A	0.738	N/A	N/A	N/A	N/A	N/A
PEU3	N/A	0.644	N/A	N/A	N/A	N/A	N/A
PEU4	N/A	0.562	N/A	N/A	N/A	N/A	N/A
PT1	N/A	N/A	0.596	N/A	N/A	N/A	N/A
PT2	N/A	N/A	0.839	N/A	N/A	N/A	N/A
TECH1	N/A	N/A	N/A	0.379	N/A	N/A	N/A
TECH2	N/A	N/A	N/A	0.791	N/A	N/A	N/A
TECH3	N/A	N/A	N/A	0.769	N/A	N/A	N/A
TECH4	N/A	N/A	N/A	0.379	N/A	N/A	N/A
TECH5	N/A	N/A	N/A	0.720	N/A	N/A	N/A
TECH6	N/A	N/A	N/A	0.764	N/A	N/A	N/A
TECH7	N/A	N/A	N/A	0.725	N/A	N/A	N/A
CUST1	N/A	N/A	N/A	N/A	0.821	N/A	N/A
CUST2	N/A	N/A	N/A	N/A	0.845	N/A	N/A
CUST3	N/A	N/A	N/A	N/A	0.411	N/A	N/A
CUST4	N/A	N/A	N/A	N/A	0.301	N/A	N/A
INT1	N/A	N/A	N/A	N/A	N/A	0.752	N/A
INT2	N/A	N/A	N/A	N/A	N/A	0.783	N/A
INT3	N/A	N/A	N/A	N/A	N/A	0.596	N/A
Message1	N/A	N/A	N/A	N/A	N/A	N/A	0.723
Message2	N/A	N/A	N/A	N/A	N/A	N/A	0.735
Message3	N/A	N/A	N/A	N/A	N/A	N/A	0.583
Message4	N/A	N/A	N/A	N/A	N/A	N/A	0.536
Message5	N/A	N/A	N/A	N/A	N/A	N/A	0.500

^a^Rotation converged in 7 iterations.

^b^Extraction method: maximum likelihood; rotation method: Promax with Kaiser normalization.

^c^PU: perceived usefulness.

^d^PEU: perceived ease of use.

^e^PT: perceived trust.

^f^TECH: technology characteristics.

^g^CUST: customization.

^h^INT: intention to use.

^i^Message: gain- and loss- framed message.

^j^N/A: not applicable.

**Table 3:** Cronbach alpha, composite reliability, and average variance extracted for the constructs.

Constructs and items		CA^a^	CR^b^	AVE^c^	Factor loading
**PEU^d^**		**.83**	**0.69**	**0.53**	
	PEU1				0.76
	PEU2				0.69
	PEU3				0.80
	PEU4				0.78
**PU^e^**		**.80**	**0.80**	**0.66**	
	PU1				0.80
	PU2				0.83
**CUST^f^**		**.82**	**0.82**	**0.70**	
	CUST1				0.78
	CUST2				0.89
	CUST3				0.49
	CUST4				0.44
**PT^g^**		**.76**	**0.71**	**0.55**	
	PT1				0.78
	PT2				0.69
**TECH^h^**		**.75**	**0.71**	**0.38**	
	TECH1				0.60
	TECH2				0.63
	TECH3				0.68
	TECH4				0.55
	TECH5				0.63
	TECH6				0.55
	TECH7				0.43
**Message^i^**		**.73**	**0.75**	**0.50**	
	Message1				0.81
	Message2				0.72
	Message3				0.57
	Message4				0.53
	Message5				0.48
**INT^j^**		**.76**	**0.77**	**0.62**	
	INT1				0.82
	INT2				0.76
	INT3				0.57

^a^CA: Cronbach alpha.

^b^CR: composite reliability.

^c^AVE: average variance extracted.

^d^PEU: perceived ease of use.

^e^PU: perceived usefulness.

^f^CUST: customization.

^g^PT: perceived trust.

^h^TECH: technology characteristics.

^i^Message: gain-loss framed message.

^j^INT: intention to use.

**Table 4:** Discriminant validity.

Factors^a^	PU^b^	PEU^c^	PT^d^	TECH^e^	CUST^f^	INT^g^	Message^h^
PU	*0.81*	N/A^i^	N/A	N/A	N/A	N/A	N/A
PEU	0.72^j^	*0.73*	N/A	N/A	N/A	N/A	N/A
PT	0.50^j^	0.46^j^	*0.74*	N/A	N/A	N/A	N/A
TECH	0.57^j^	0.66^j^	0.59^j^	*0.62*	N/A	N/A	N/A
CUST	0.29^j^	0.27^j^	0.36^j^	0.32^j^	*0.84*	N/A	N/A
INT	0.74^j^	0.50^j^	0.41^j^	0.58^j^	0.27^j^	*0.79*	N/A
Message	−0.09	−0.11	−0.03	−0.07	−0.001	0.02	*0.71*

^a^Off-diagonal elements are correlations, and diagonal elements are square roots of the average variance extracted.

^b^PU: perceived usefulness.

^c^PEU: perceived ease of use.

^d^PT: perceived trust.

^e^TECH: technology characteristics.

^f^CUST: customization.

^g^INT: intention to use.

^h^Message: gain-loss framed message.

^i^N/A: not applicable.

^j^0.27: significance of correlations *P*<.001.

### Structural Equation Modeling

In the second stage, CFA was conducted before testing the hypothesized relationships among the constructs in smart health awareness message framework using SEM [83]. To proceed with CFA, standardized loadings for each item were obtained, in which these values should be at least 0.5 or ideally 0.7 or higher [78]. As shown in Table 5, of the 27 items, CUST3, CUST4, TECH7, and Message5 are attributed to deletion from the research model because of their lower loadings, whereas others are well related to their associated constructs. Given the significant standardized residual covariances, which means the largest values (in absolute value) for items PEU3, PEU4, TECH5, TECH6, Message4, and INT3, they require removal as they affect the goodness fit of the model [84] (Multimedia Appendix 4). The analysis illustrated in Multimedia Appendix 5 confirmed that the linear regression model is adequately fit, with χ^2^_104_ value of 299.0 and *P*<.001.

In the second step of the CFA, model fit indexes were measured: χ^2^ divided by *df*, root mean square error of approximation (RMSEA), normed fit index (NFI), incremental fit index (IFI), comparative fit index (CFI), and Tucker-Lewis Index (TLI) [83]. The CFA results showed an acceptable fit model (χ^2^_97_=145.8; χ^2^ divided by *df*=1.503; RMSEA=0.036; NFI=0.937; IFI=0.978; CFI=0.978; TLI=0.969; Multimedia Appendix 6). The results also confirm that the linear and covariance fit models meet the standards, thus emphasizing the acceptance of model fit (Multimedia Appendices 7 and 8, respectively).

The next step is measuring the path coefficient, coefficient of determination, and *t* value using SEM. A path coefficient or path analysis indicates the relationships among the constructs. The coefficient of determination (R2) is a measure of the percentage of the total variation of the dependent variable that is explained or predicted by the independent variable(s) or predictor(s) [78]. The larger the value of the coefficient of determination, the greater the prediction of the dependent variable. Table 5 illustrates the path analysis and hypotheses testing. The results show that the coefficient of determination (R2) is 0.642 for the *intention to use* construct. This means that the 4 constructs (perceived ease of use, perceived usefulness, perceived trust, gain-framed message, and loss-framed message) moderately explain 64.2% of the variance in intention to use social media. Perceived ease of use, together with customization, perceived trust, and technology characteristics, explain 55.4% of the variance in perceived usefulness. Finally, customization explains 26.2% of the variance in perceived ease of use.

**Table 5:** Summary of testing hypotheses.

Hypothesis	Hypothesized path	Beta^a^	*P* value	Result
H1	PEU^b^-INT^c^	.05	.43	Not supported
H2	PEU-PU^d^	.37	<.001	Supported
H3	PU-INT	.43	<.001	Supported
H4	CUST^e^-PEU	.12	.12	Not supported
H5	CUST-PU	.16	.05	Supported
H6	PT^f^-INT	.11	.08	Not supported
H7	PT-PU	.07	<.001	Supported
H8	TECH^g^-PU	.12	<.001	Supported
H9	Gain-framed message-INT	.04	<.001	Supported
H10	Loss-framed message-INT	.08	<.001	Supported

^a^Beta is standardized.

^b^PEU: perceived ease of use.

^c^INT: intention to use.

^d^PU: perceived usefulness.

^e^CUST: customization.

^f^PT: perceived trust.

^g^TECH: technology characteristics.

Path analysis results also reveal that perceived ease of usefulness has little effect on intention to use (β=.05; *P*=.43), unlike the significant effect on perceived usefulness (β=.37; *P*<.001). Thus, H1 is not supported, whereas H2 is supported. The impact of perceived usefulness on intention to use is significant (β=.43; *P*<.001), supporting H3. However, the results indicate that customization has no significant impact on perceived ease of use (β=.12; *P*=.12), whereas it has a significant impact on perceived usefulness (β=.16; *P*=.05). Hence, H4 is not supported, and H5 is supported. The results also indicate that there is no significant impact between perceived trust and intention to use (β=.11; *P*=.07). In contrast, perceived trust significantly impacts perceived usefulness (β=.07; *P*<.001). Therefore, H6 is not supported, whereas H7 is supported. Technology characteristics are considered to be related and have a significant impact on perceived usefulness (β=.12; *P*<.001), lending support to H8. Finally, it was found that gain-framed messages and loss-framed messages are significantly and positively related to intention to use social media (β=.04; *P*<.001) and (β=.08; *P*<.001), respectively. Thus, H9 and H10 are supported.

## Discussion

### Principal Findings

**Figure 2:**
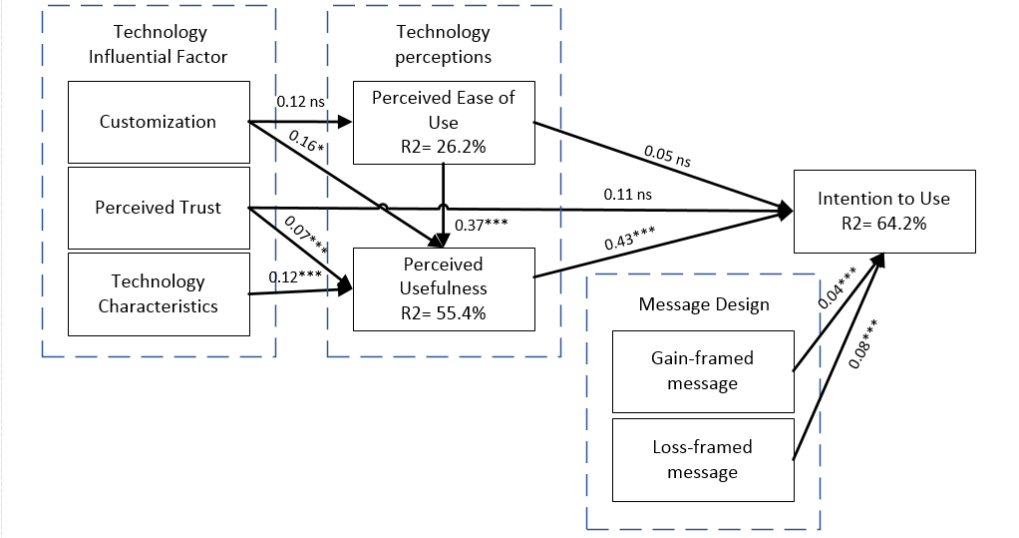
Hypothesized Smart Health Awareness Message Framework. *P<.05, **P<.01, ***P<.001; ns: nonsignificant.

Nowadays, social media plays a considerable role in an individual’s daily routine, as it provides different features that encourage people to adapt it for a range of uses, including health promotion. Therefore, the motivation of this paper was to examine the factors that affect people’s intention to use social media as a way of receiving health awareness messages, which, in turn, will help them to maintain their diet and reduce the incidence of diseases. In turn, the challenges that arise from printed media, involving paper and power consumption, storage capacity, and labor intensity, will be reduced. The results in Table 1 show that 69.0% (270/ 391) of the public surveyed used social media always, often, or very often, and 31.0% (121/391) of them hardly or never used social media. This indicates that eHealth that involves using social media to convey health messages has the potential to reach about 70% of the public. Findings (shown in Figure 2) confirm that perceived usefulness and message design relating to health message frames (positive vs negative) are the leading predictors of people’s intention to use social media in the health promotion context. Loss-framed messages have been examined in previous studies [85,86] to be a motivating factor that influences people to engage and comply with health behavior on social media. A recent study has come to an opposing conclusion where people are encouraged to acquire and trust health information on social media when they are exposed to gain-framed messages [87]. Regarding social media usefulness, the results of this study are consistent with those of Lin and Ho [49], indicating that perceived usefulness significantly affects people toward social media adaptation in sharing health information.

In addition, health message customization encourages the prediction of perceived usefulness, whereas it has no effect on perceived ease of use of social media. Thus, it can be indicated that social media users perceive the acquired benefits from social media when they receive health messages tailored to their preferences [51]. Regarding the relationship between technology characteristics and perceived usefulness (β=.12; *P*<.001), it can be concluded that the higher the characteristics offered by social media, the greater the perceptions of the benefits of adopting health messages received via social media. These characteristics involve hyperlinks and hashtags provision, which, in turn, generate higher engagement with messages [88]. Moreover, posting photographs and videos that enhance the message by being more visual encourage individuals to adopt social media for health promotion [15]. The results reveal that the design of health messages plays a significant role in people’s intention to use social media. Consequently, the key factors specified are essential for health organizations to promote eHealth by developing and spreading health messages effectively, which, in turn, will enhance people’s health.

### Conclusions

The study’s results demonstrate the use of social media in health promotion purposes, which will enhance the outcomes of an individual’s well-being. This paper aimed to investigate the influential factors that affect people’s intention to adopt such technology in health communication campaigns. Undoubtedly, high levels of health message success cannot be achieved without emotions embedded in the content of health messages [89]. The study’s findings indicate that health message frames would be efficacious in improving public health communication toward social media adaptation. Furthermore, perceived usefulness has an impact on people’s intention to adapt to social media to acquire health awareness information. These results can be explained by the adaptation of the TAM and the prospect theory.

Given the findings of smart health awareness message framework, designing health awareness messages to include loss- or gain-framed content to evoke high emotions might contribute to boosting the effectiveness of health promotion interventions. Hence, this study offers implications for health awareness message developers that guide them to establish materials that are more patient friendly and technologically outstanding by adapting social media as a delivery method. Accordingly, this strategy will encourage individuals to exchange these messages among social media users.

### Limitations and Future Work

This study has several limitations and indicates several directions for future work. First, for the construct of message design, there are few studies associated with the prospect theory that examine the public perspective in terms of their preferences. Thus, the authors developed a number of items, validated by experts, and adapted in this study to ensure the validity of the construct. Future works might examine this construct more broadly to determine the extent to which the public might receive this message in a more positive or negative manner. Second, although the study involved 391 respondents from different countries, in which sample size is convenient for testing the framework, future studies with larger samples are needed to reinforce the generalization of results. In addition, the participants were English speakers, and findings related to a particular language might restrict generalization to others. Thus, future research might duplicate this study with different languages.

Smart health awareness message framework will also be used to define the right content and format of the health awareness messages to be spread via a software system that is integrated with different social media platforms. Furthermore, a computer-based knowledge framework based on the use of social media apps will be developed to spread health awareness messages. Finally, a specific statistical technique will be used to validate the impact of the health awareness message on recipients.

## Appendix

Multimedia Appendix 1 Items of the study’s constructs.

Multimedia Appendix 2 Items correlation matrix.

Multimedia Appendix 3 Kaiser-Meyer-Olkin and Bartlett test.

Multimedia Appendix 4 Standardized residual covariances for deleted items.

Multimedia Appendix 5 Standardized estimate of linear regression.

Multimedia Appendix 6 Model fit summary.

Multimedia Appendix 7 Covariance fit model.

Multimedia Appendix 8 Standardized regression weights among items.

## Abbreviations

AVE: average variance extracted
CA: Cronbach alpha
CFA: confirmatory factor analysis
CFI: comparative fit index
CR: composite reliability
EFA: exploratory factor analysis
eHealth: electronic health
IFI: incremental fit index
KMO: Kaiser-Meyer-Olkin
NFI: normed fit index
RMSEA: root mean square error of approximation
SEM: structural equation modeling
TAM: technology acceptance model
TLI: Tucker-Lewis Index
TTF: task technology fit
