# Supplementary material for: The Development of a Smart Health Awareness Message Framework Based on the Use of Social Media: Quantitative Study
Source: J Med Internet Res. 2020 Jul 23;22(7):e16212. doi: 10.2196/16212 (PMC7413284; doi:10.2196/16212)
Supplement: Multimedia Appendix 1 [file jmir_v22i7e16212_app1.docx]

Multimedia Appendix 1

**Items of study’s constructs**

| Construct | Code | Item | Mean | Standard Deviation | Reference |
| --- | --- | --- | --- | --- | --- |
| Perceived Ease of Use | PEU | 1. Learning how to use social media to adopt health awareness message is easy for me. 2. My interaction with social media to adopt health awareness message is clear and Understandable. 3. I find using social media to adopt health awareness message easy to use. 4. It is easy for me to become skilful at using social media to adopt health awareness message. | 3.77  3.63  3.75  3.60 | 0.944  0.853  0.899  0.903 | [74] |
| Perceived Usefulness | PU | 1. I expect that using social media for adopting health awareness messages will give me the necessary medical information. 2. I believe that using social media for adopting health awareness messages will save me a lot of time. | 3.34  3.53 | 1.045  1.025 | [75] |
| Customisation | CUST | 1. I like health awareness message customized to my local time. 2. I like health awareness message customized to my location. 3. I prefer health awareness message customized based on my health situation. 4. I prefer health awareness message customized based on social media platform I like. | 3.61  3.60  3.45  3.40 | 0.881  0.914  0.901  0.857 | [65] |
| Perceived Trust | PT | 1. Based on my experience with healthcare providers I know they are trustworthy. 2. I expect that health organizations which spread health awareness messages will provide trustful information. | 3.81  3.56 | 0.899  0.848 | [75] |
| Technology Characteristics | TECH | 1. The number of characters design of social media platforms is adequate for obtaining health information. 2. When I use social media, I can easily recognize where the needed information is located. 3. When I use social media, I can easily recognize where I navigate. 4. I think that the screen design of social media is harmonious. 5. Health awareness messages available social media must provide relevant pictures. 6. Health awareness messages available social media must provide relevant animation. 7. Health awareness messages available social media must provide relevant hyperlinks. | 3.21  3.46  3.57  3.37  3.91  3.76  3.92 | 0.822  0.940  0.883  0.773  0.816  0.870  0.823 | [65,76] |
| Gain- Loss framed Message | Message | 1. Using sunscreen can keep your skin healthy. 2. Not using sunscreen can cause skin cancer. 3. Drinking a lot of water daily can help you lose weight. 4. Not drinking enough water causes you gain weight. 5. Messages include information about diseases such as symptoms, treatments and prevention methods. | 3.38  3.42  3.59  3.24  3.34 | 0.859  0.852  0.959  1.069  0.974 | Health care experts |
| Intention To Use | INT | 1. I intend to continue using social media to adopt health awareness messages in the future. 2. I will always try to use social media to adopt health awareness messages in my daily life. 3. I will keep using social media to adopt health awareness messages as regularly as I do now. | 3.61  3.42  3.34 | 0.884  0.973  0.807 | [74] |
