# Supplementary figures and images for: The Development of a Smart Health Awareness Message Framework Based on the Use of Social Media: Quantitative Study
Source: J Med Internet Res. 2020 Jul 23;22(7):e16212. doi: 10.2196/16212 (PMC7413284; doi:10.2196/16212)

Multimedia Appendix 2 **Items Correlation Matrix**


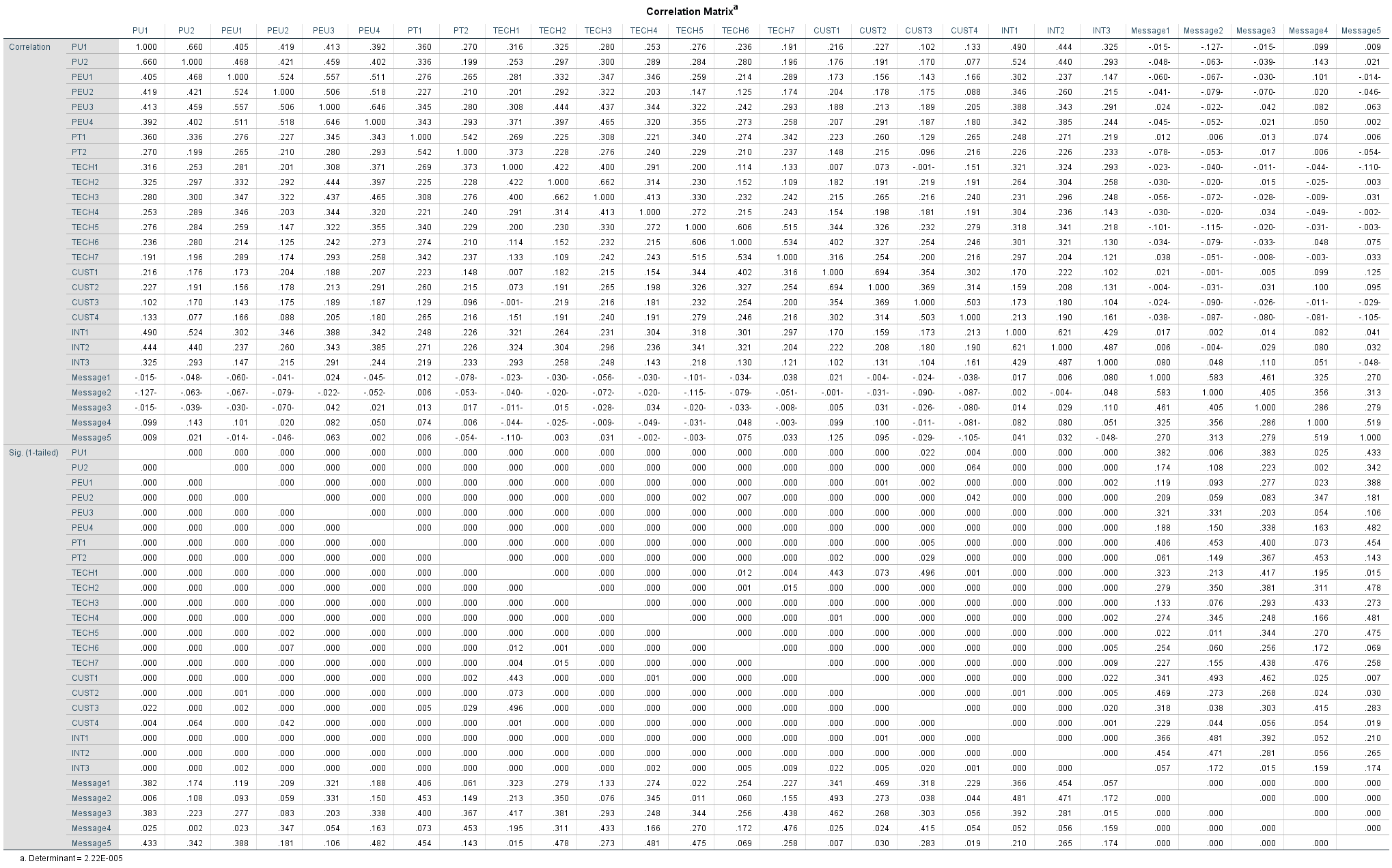

Supplement: Multimedia Appendix 2 [file jmir_v22i7e16212_app2.docx]

Multimedia Appendix 4

**Standardized Residual Covariances (SRCs) for Deleted Items**


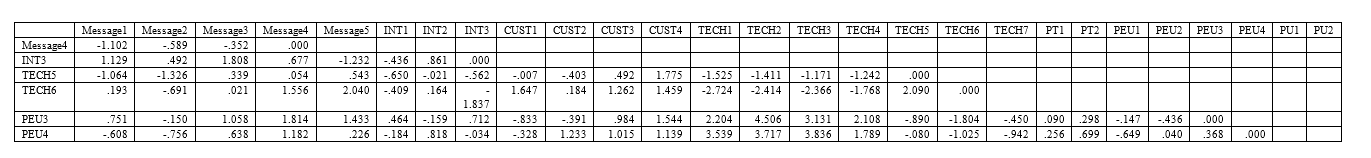

Supplement: Multimedia Appendix 4 [file jmir_v22i7e16212_app4.docx]
