# Supplementary material for: The Development of a Smart Health Awareness Message Framework Based on the Use of Social Media: Quantitative Study
Source: J Med Internet Res. 2020 Jul 23;22(7):e16212. doi: 10.2196/16212 (PMC7413284; doi:10.2196/16212)
Supplement: Multimedia Appendix 3 [file jmir_v22i7e16212_app3.docx]

Multimedia Appendix 3

**KMO and Bartlett's Test**

|  | | |
| --- | --- | --- |
| Kaiser-Meyer-Olkin Measure of Sampling Adequacy. | | .805 |
| Bartlett's Test of Sphericity | Approx. Chi-Square | 2466.994 |
|  | Df | 190 |
|  | Sig. | .000 |
