# Supplementary material for: The Development of a Smart Health Awareness Message Framework Based on the Use of Social Media: Quantitative Study
Source: J Med Internet Res. 2020 Jul 23;22(7):e16212. doi: 10.2196/16212 (PMC7413284; doi:10.2196/16212)
Supplement: Multimedia Appendix 5 [file jmir_v22i7e16212_app5.docx]

Multimedia Appendix 5

**Linear Regression (Standardized Estimate)**


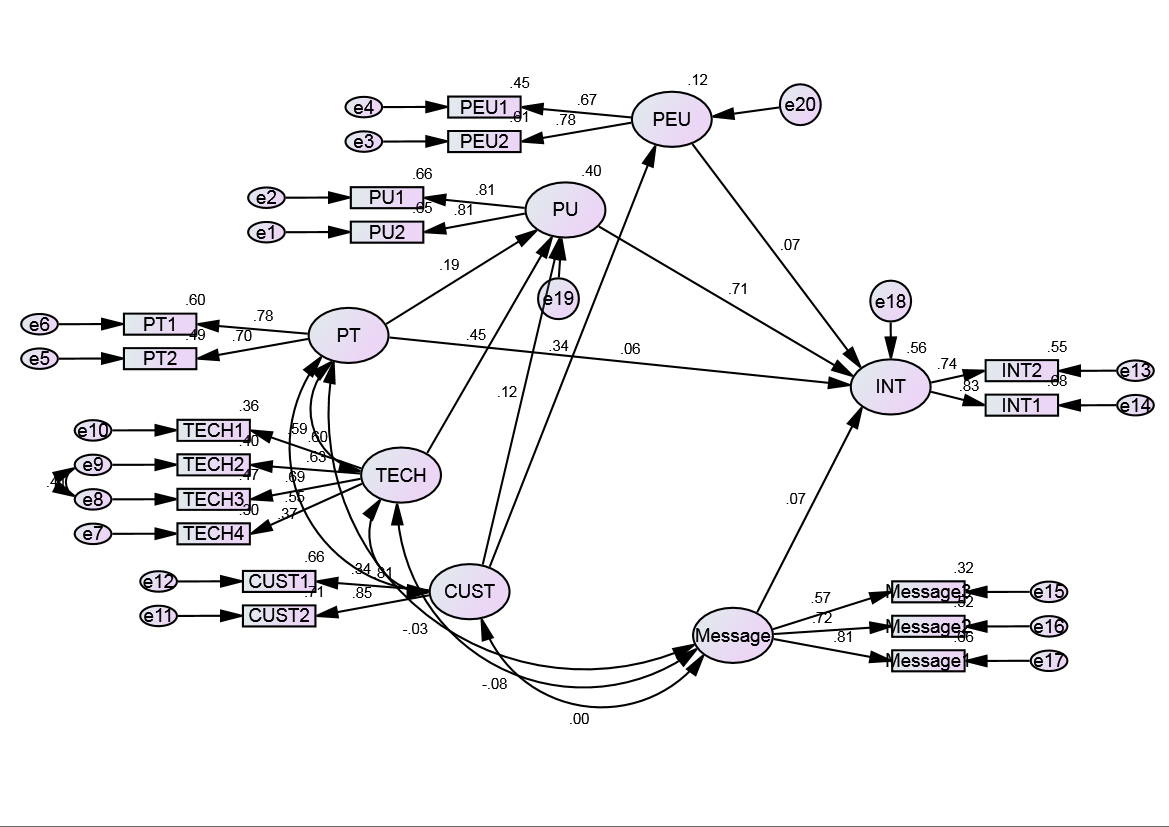


Note: Minimum was achieved

Chi-square = 299.052

Degrees of freedom = 104

Probability level = .000

**Covariances (Group number 1 - Default model)**

|  |  |  | Estimate | S.E. | C.R. | P | Label |
| --- | --- | --- | --- | --- | --- | --- | --- |
| PT | <--> | TECH | .147 | .028 | 5.340 | *** | par_20 |
| PT | <--> | CUST | .171 | .034 | 4.948 | *** | par_21 |
| Message | <--> | PT | -.011 | .022 | -.476 | .634 | par_22 |
| TECH | <--> | CUST | .112 | .026 | 4.242 | *** | par_23 |
| Message | <--> | TECH | -.017 | .016 | -1.104 | .270 | par_24 |
| Message | <--> | CUST | -.002 | .027 | -.074 | .941 | par_25 |
| e8 | <--> | e9 | .191 | .043 | 4.434 | *** | par_11 |
