# Supplementary material for: The Development of a Smart Health Awareness Message Framework Based on the Use of Social Media: Quantitative Study
Source: J Med Internet Res. 2020 Jul 23;22(7):e16212. doi: 10.2196/16212 (PMC7413284; doi:10.2196/16212)
Supplement: Multimedia Appendix 6 [file jmir_v22i7e16212_app6.docx]

Multimedia Appendix 6

**Model Fit Summary**

| **Measure** | **Estimate** | **Threshold** | **Interpretation** |
| --- | --- | --- | --- |
| **CMIN** | 145.786 | -- | -- |
| **DF** | 97.000 | -- | -- |
| **CMIN/DF** | 1.503 | Between 1 and 3 | Excellent |
| **CFI** | 0.978 | >0.95 | Excellent |
| **SRMR** | 0.035 | <0.08 | Excellent |
| **RMSEA** | 0.036 | <0.06 | Excellent |
| **PClose** | 0.979 | >0.05 | Excellent |

Note: CMIN= Minimum discrepancy; CFI= Comparative Fit Index; SRMR= Standardized Root Mean Square Residual; RMSEA= Root Mean Square Error of Approximation; PClose= Probability of RMSEA

Congratulations, your model fit is excellent.
