# Supplementary material for: The Development of a Smart Health Awareness Message Framework Based on the Use of Social Media: Quantitative Study
Source: J Med Internet Res. 2020 Jul 23;22(7):e16212. doi: 10.2196/16212 (PMC7413284; doi:10.2196/16212)
Supplement: Multimedia Appendix 7 [file jmir_v22i7e16212_app7.docx]

Multimedia Appendix 7

**Covariance Fit Model**

|  |  |  | Estimate | S.E. | C.R. | P |
| --- | --- | --- | --- | --- | --- | --- |
| PU | <--> | PEU | .357 | .046 | 7.841 | 0.000 |
| PU | <--> | PT | .246 | .039 | 6.364 | 0.000 |
| PU | <--> | TECH | .207 | .033 | 6.268 | 0.000 |
| PU | <--> | CUST | .201 | .044 | 4.532 | 0.000 |
| PU | <--> | Message | -.039 | .030 | -1.303 | .193 |
| PEU | <--> | PT | .157 | .030 | 5.208 | 0.000 |
| PEU | <--> | TECH | .164 | .026 | 6.274 | 0.000 |
| PEU | <--> | CUST | .130 | .034 | 3.884 | 0.000 |
| PEU | <--> | Message | -.034 | .022 | -1.519 | .129 |
| PT | <--> | TECH | .147 | .027 | 5.388 | 0.000 |
| PT | <--> | CUST | .169 | .036 | 4.751 | 0.000 |
| PT | <--> | Message | -.009 | .022 | -.393 | .694 |
| TECH | <--> | CUST | .111 | .027 | 4.031 | 0.000 |
| TECH | <--> | Message | -.016 | .016 | -.971 | .332 |
| CUST | <--> | Message | -.001 | .028 | -.022 | .982 |
| PU | <--> | INT | .461 | .053 | 8.722 | 0.000 |
| PEU | <--> | INT | .214 | .036 | 5.915 | 0.000 |
| PT | <--> | INT | .178 | .035 | 5.069 | 0.000 |
| TECH | <--> | INT | .182 | .031 | 5.955 | 0.000 |
| CUST | <--> | INT | .161 | .040 | 4.011 | 0.000 |
| INT | <--> | Message | .007 | .026 | .258 | .796 |

Note: The Critical Ratio (CR) is calculated by dividing the parameter estimate by its Standard Error (SE).
