# Supplementary material for: The Development of a Smart Health Awareness Message Framework Based on the Use of Social Media: Quantitative Study
Source: J Med Internet Res. 2020 Jul 23;22(7):e16212. doi: 10.2196/16212 (PMC7413284; doi:10.2196/16212)
Supplement: Multimedia Appendix 8 [file jmir_v22i7e16212_app8.docx]

Multimedia Appendix 8

**Standardised Regression Weights among Items**

|  | | | Estimate | S.E. | C.R. | P | Standardized regression weights |
| --- | --- | --- | --- | --- | --- | --- | --- |
| PU2 | <--- | PU | 0.826 | 0.066 | 15.037 | p<0.001 | 0.799 |
| PU1 | <--- | PU | 0.799 | 0.121 | 10.223 | p<0.001 | 0.764 |
| PEU2 | <--- | PEU | 0.685 | 0.133 | 7.191 | p<0.001 | 0.693 |
| PEU1 | <--- | PEU | 0.764 | 0.146 | 8.191 | p<0.001 | 0.781 |
| PT2 | <--- | PT | 0.693 | 0.146 | 6.818 | p<0.001 | 0.555 |
| PT1 | <--- | PT | 0.781 | 0.166 | 8.488 | p<0.001 | 0.683 |
| TECH4 | <--- | TECH | 0.555 | 0.176 | 7.818 | p<0.001 | 0.627 |
| TECH3 | <--- | TECH | 0.683 | 0.148 | 7.721 | p<0.001 | 0.598 |
| TECH2 | <--- | TECH | 0.627 | 0.144 | 7.724 | p<0.001 | 0.891 |
| TECH1 | <--- | TECH | 0.598 | 0.120 | 7.023 | p<0.001 | 0.779 |
| CUST2 | <--- | CUST | 0.891 | 0.174 | 9.714 | p<0.001 | 0.755 |
| CUST1 | <--- | CUST | 0.779 | 0.080 | 12.292 | p<0.001 | 0.822 |
| INT2 | <--- | INT | 0.755 | 0.088 | 11.292 | p<0.001 | 0.568 |
| INT1 | <--- | INT | 0.822 | 0.124 | 9.119 | p<0.001 | 0.723 |
| Message3 | <--- | Message | 0.568 | 0.143 | 8.901 | p<0.001 | 0.806 |
| Message2 | <--- | Message | 0.723 | 0.125 | 10.319 | p<0.001 | 0.578 |
| Message1 | <--- | Message | 0.806 | 0.173 | 8.931 | p<0.001 | 0.850 |
